# Baby food industry interference with infant feeding international regulation—A case study on the standard for follow-up formula

**DOI:** 10.3389/fpubh.2022.984385

**Published:** 2022-11-22

**Authors:** Kimielle Cristina Silva, Inês Rugani Ribeiro de Castro, Camila Maranha Paes de Carvalho, Kenneth Rochel de Camargo

**Affiliations:** ^1^Institute of Social Medicine, State University of Rio de Janeiro (UERJ), Rio de Janeiro, Brazil; ^2^Institute of Nutrition, State University of Rio de Janeiro (UERJ), Rio de Janeiro, Brazil; ^3^Department of Social Nutrition, Faculty of Nutrition, Fluminense Federal University (UFF), Rio de Janeiro, Brazil

**Keywords:** conflict of interest, commercial determinants of health, infants and young children feeding, breastmilk substitutes, baby food industry, infant formula

## Abstract

**Introduction:**

Globally, first-food systems have changed and breastfeeding has decreased due to the increased growth in commercial breast milk substitute (BMS) consumption, which includes both follow-up and toddler formulas. These products are manufactured by a small number of corporate leaders in international BMS sales. Discussions for global regulation of these products take place in the Codex Alimentarius and are permeated by the strong participation of these corporations in the Codex committees.

**Objective:**

In the present study, the participation of the baby food industry in the review of the follow-up formula standard in the Codex Committee on Nutrition and Foods for Special Dietary Uses (CCNFSDU) was analyzed.

**Methods:**

The analysis of the CCNFSDU documents was based on the period from 2009 to 2019 and used quantitative and qualitative approaches. Compositional and participation data from country delegations and observer organizations on the representative profiles of the involved institutions and the baby food industry's involvement in this process were established systematically.

**Results:**

In total, 134 out of the 189 Codex Alimentarius member countries engaged in the standard review process, of which 28% were involved in the entire process. The private sector was present in 81% of the most assiduous member state delegations to the meetings. Furthermore, ~60% of the observer organizations involved in the review process were business associations representing industry interests. Moreover, the International Special Dietary Foods Industries was the only business association with observer status in the CCNFSDU that was specifically dedicated to representing the baby food industryduring the review process.

**Conclusion:**

These research results expand the body of evidence confirming the expressive and disproportionate participation of baby food industries and their representatives in the discussion processes within the scope of the CCNFSDU. However, studies investigating the Codex and the public documents of its respective committees are limited. Thus, this was the first study to analyze the influence of the baby food industry on BMS global regulatory compliance.

## Introduction

“Codex Alimentarius” refers to a set of rules, guidelines, and codes of practice coordinated by the Codex Alimentarius Commission (CAC). The CAC is responsible for all matters regarding the implementation of the Joint Food and Agricultural Organization (FAO)/World Health Organization (WHO) Food Standard Program. All member countries and FAO/WHO affiliate members are eligible to join the Commission, in addition to scientific association observers, industries, food businesses, and consumers. These food standards and related texts aim at protecting consumers' health and ensuring fair practices in the food trade (1). While the process to establish standards and guidelines is complex, it is designed to enable access to a wide range of stakeholders (2). Countries are allowed to create their own standards for public health, food security, and nutrition. Thus, the Codex regulations influence the national regulatory processes to ensure global food safety. However, evidence shows that standards facilitate business, prioritize trade concerns, and align with the interests of agri-food companies and the food industry, rather than adhering to health and food security (3–11).

Several studies have reported the influence of the food industry on the Codex (5, 7–13), primarily concerning committees for infant formulas, labeling, and additives. All of these committees benefit from the active involvement of observer organizations, including business associations from several pharmaceutical and food industries. In the context of products for infant feeding, particularly follow-up formulas, the first global standard for product compliance was adopted in 1987. The draft proposal for the follow-up milk standard by the Swiss delegation was submitted in 1975 and the Codex Commission approved it 12 years later in 1987. Thereafter, changes in product amendments and nomenclature were implemented until a consensus was reached for the name of the follow-up formula in 1987 (14).

Following the publication of the follow-up formula standard, a considerable amount of evidence regarding baby food and nutrition found widespread changes in the global food system, such as steep declines in breastfeeding and the normalization of formula feeding in many countries. In 2010, the New Zealand delegation proposed the preparation of a discussion article for the Codex Committee on Nutrition and Foods for Special Dietary Uses (CCNFSDU) to review the standard for follow-up formula. To date (i.e., November 2022), the review process has begun but is yet to be finalized.

“Non-government organizations that represented the baby food industry and other business interests attended and presented their perspectives throughout all the steps of the standard definition process with the CCNFSDU. According to Baker et al. (13), the “baby food industry” compasses Big Formula, the dairy industry as well as other input suppliers, retailers, advertising companies, and several other commercial entities profiting from the breast milk substitute (BMS).

Big Formula includes a limited number of corporations originating from food and pharmaceutical industries in Europe and the United States of America (USA) that dominate infant formulas. Furthermore, they manufacture major brands available on the market (13, 15). Big Formula comprises a network of trade associations and other influential organizations with corporate funding (e.g., lobbying groups and advertising associations). These organizations safeguard the interests of Big Formula on a global level and promote favorable regulatory environments for the expansion of their products (13). Moreover, some of these groups and associations have observer organization status on the Codex.

A coordinated network of commercial associations and Big Formula actively engages in political scenarios and regulatory arenas concerning baby and early childhood foods that influence the sales of these industries at a global level, such as the WHO, Codex Alimentarius, and the World Trade Organization (WTO) (13). In the context of debates about commercial determinants of health are strategies and approaches used by the private sector to promote products and alternative options. Thus, a special investigation into the commercial determinants of maternal, infant, and young child health has been conducted (13, 16). Investigating the corporate activities of the commercial actors specific to this context is pertinent to understand the corporate power that shapes first-food systems, fosters infant formula use and promotion, and jeopardizes breastfeeding on a global scale (8, 13, 15, 17–26). Participation in decision-making processes is one of these corporate actions. This study analyzed the participation of the baby food industry in the decision-making process regarding the revision of the standard for follow-up formula at the Codex Committee on Nutrition and Foods for Special Dietary Uses (CCNFSDU).

## Materials and methods

In this study, an exploratory analysis was performed by conducting a case study of the standard for the follow-up formula review process from 2009 to 2019. According to Yin (27), the strategy of implementing a case study is applicable when the researcher has no control of the events and focus is placed on facts concerning a specific real-life context, aiming to understand complex social phenomena.

This study systematized the composition of the delegations of member states and observer organizations and their attendance at meetings, the profiles of the participants from these institutions, and the extent of participation of the baby food industry and other industry groups in the revision process of the Codex standard for follow-up formula. Documents on the subject were collected from the Codex Alimentarius website on the CCNFSDU-specific page, where all documents available for public access can be found (https://www.fao.org/fao-who-codexalimentarius/en/). Data collection was performed in January 2020.

Collected documents included (a) CCNFSDU session reports, called ALINORM and REP (Commission, Committees, and Work Groups reports, as well as work documents for CAC session periods) and (b) Electronic Working Groups (EWG) and Physical Working Group (PWG) and Committee (referred to as CX) work documents, and Conference Room Documents (CRD). The collected and analyzed documents are listed in Supplementary material.

### Data extraction and organization

A list of participants by member state delegation and observer organization is at the end of each ALINORM and REP, where the individual information of each session participant is entered. Information regarding session number and year, representative delegation, and the representative institution, industry, or country was collected and organized. All types of observer organizations and the participant's country of origin were registered. Participants from member state delegations and observer organizations were categorized into government, business associations, scientific organizations, human rights protection organizations (i.e., maternal and children's rights and breastfeeding), as well as consumer, humanitarian, and intergovernmental organizations.

### Analysis of participating members

In total, 11 sessions were held during the study period and an Excel spreadsheet was used to count each session attendance of the participating member states. Based on these data, four group categories were created. That is, Group A comprised member states attending at least nine sessions; Group B comprised member states attending six to eight sessions; Group C comprised members states attending three to five sessions; and Group D comprised member states attending up to two sessions. Table 1 presents a description of the analysis methods used to measure delegation participation from member countries and observer organizations.

**Table 1:** Description of the methods adopted to analyze CCNFSDU^a^ participation.

**Analysis**	**Method**
Participation percentage by group^b^	Number of participants in each group divided by the total number of participants
Participation percentage by group and by the *Codex Alimentarius* Regions	Countries were grouped according to the *Codex* geographic regions (Africa, Asia, Europe, Latin America and Caribbean, North America and South West Pacific, and the Middle East), and each region percentage was calculated based on the group
Private interest participants' percentage in member countries delegations	Private interest participants' rate per year was calculated for each country. Moreover, the average ratio for the years analyzed and aggregate private interest rates for participants were calculated, considering each country investigated during the second year
Total participants by interest group	Private interest (commercial associations participants and food industry) and public interest (government sectors representatives, universities, scientific associations, defense of rights associations, humanitarian organizations, and intergovernmental and consumer organizations) participants were categorized. Absolute and relative frequencies were calculated for each of the groups per year studied. These values were also averaged for the total number of years studied
Actors' participation percentage by group^b^	Each actor's group percentage was calculated according to the four group categories of attendance at the 11 sessions that took place during the period. The software Adobe^®^ Illustrator version 25.2.3 was used for figure development. The figure was constructed in a circle layout, where each frame represented one group according to the attendance at the 11 sessions that took place during the period. Each color represented the actors involved in the process. Frames were introduced to display the number of actors in each meeting's attendance grouping

a Codex Committee on Nutrition and Foods for Special Dietary Uses.

b Group A: Members attending at least nine sessions.

Group B: Members attending 6–8 sessions.

Group C: Members attending 3–5 sessions.

Group D: Members attending two sessions.

### Analysis of participating private interest representatives

Table 2 presents the variables used in the analysis of private interest participants, including representatives from business associations and the baby food industry. Data from participants attending at least seven sessions were used (i.e., members who attended more than half of the sessions held). The private interest of each participant is shown using a line that indicates the representative institution or industry, country of origin, total years of attendance, and the delegation that the member was representing each year.

**Table 2:** Variables applied in private interest participants analysis, CCNFSDU^a^, 2009–2019.

**Variable**	**Description**
Actors	Participant name initials
Origin	Participant country of origin
Total years	Total number of participants attending CCNFSDU sessions from 2009 to 2019
Delegation Country industry	The participant is part of the member state delegation, but represents a specific company
Delegation Country business association	The participant is part of the member country delegation, but represents an association that defends the interests of industries
Delegation Country University	It is part of member state delegation as specialized support
Observer *Codex*	It is part of an observer organization accredited by the *Codex*
Observer Codex_Industry	It is part of the observer organization delegation accredited by the *Codex*, but communicated that it represents food industry interests

a The Codex Committee on Nutrition and Foods for Special Dietary Uses.

### Link analysis between International Special Dietary Foods Industries and the baby food industry

The International Special Dietary Foods Industries (ISDI) website was analyzed to assess the links between this observer organization and the baby food industry. Thereafter, information was collected on the constituting associations. Data were extracted in November 2020 and were systematized using an Excel worksheet.

These links are expressed in figures exhibiting the four main Big Formula companies which include the transnational formula manufacturers that account for 55% of the formulas for infants and young children in the global market, namely, Nestlé, Danone, MeadJohnson, and Abbott (15). The links between transnational corporations that control the global production and distribution of ultra-processed products have also been presented. Furthermore, each business association member of the ISDI as well as the accompanying industry was identified. Each circle represents a transactional formula manufacturer with a color assigned to each circle. Lines with the same circle color were used to indicate links between business associations and the Big Formula, and each business association was represented by its acronym and country of origin. Big food is represented by orange circles with link lines of the same color. The software Adobe Illustrator version 25.2.3 was used for developing the figures.

## Results

### Composition and attendance of member state delegations and observer delegations in codex standard for follow-up formula review

In 2021, the Codex Alimentarius Commission comprised 189 members, namely, 188 member states and one member organization [i.e., European Union (EU)] (28). Among these, 134 members (71%) attended at least one of the CCNFSDU sessions during the investigated period, indicating heterogeneous country attendance during this period. Notably, 28% of the member countries regularly attended the meetings (Group A), while 37% attended < 3 of the 11 meetings during the investigated period (Group D; Table 3).

**Table 3:** Member countries attending CCNFSDU^1^ according to number of sessions 2009–2019.

**Group A^a^**		**Group B^b^**		**Group C^c^**		**Group D^d^**	
***n*** = **37 (28%)**		***n*** = **20 (15%)**		***n*** = **28 (21%)**		***n*** = **49 (37%)**	
**Member country**	***N*# sessions**	**Member country**	***N*# sessions**	**Member country**	***N*# sessions**	**Member country**	***N*# sessions**
South Africa	11	Saudi Arabia	8	Bangladesh	5	Angola	2
Germany	11	Spain	8	Cameroon	5	Antigua and Barbuda	2
Australia	11	Hungary	8	Cambodia	5	Bulgaria	2
Belgium	11	Ireland	8	Ivory Coast	5	Kazakhstan	2
Brazil	11	Russia	8	Cuba	5	Gambia	2
Canada	11	Turkey	8	Ecuador	5	Yemen	2
China	11	Vietnam	8	Mali	5	Jamaica	2
Colombia	11	Algeria	7	Paraguay	5	Jordan	2
South Korea	11	Denmark	7	Peru	5	Libya	2
United States of America	11	Estonia	7	Uganda	5	Luxembourg	2
France	11	Iran	7	Burkina Faso	4	Mauritania	2
Netherlands	11	Lithuania	7	Qatar	4	Moldavia	2
India	11	Morocco	7	Tanzania	4	Burma	2
Indonesia	11	Senegal	7	Uruguay	4	Nicaragua	2
Italy	11	Togo	7	Benin	3	Rwanda	2
Japan	11	Argentina	6	Bolivia	3	Samoa	2
Kenya	11	Slovakia	6	Botswana	3	Trinidad and Tobago	2
Malaysia	11	Kuwait	6	Croatia	3	Tunisia	2
Norway	11	Nepal	6	Ethiopia	3	Armenia	1
New Zealand	11	United Kingdom	6	Greece	3	Azerbaijan	1
Sweden	11			Iraq	3	Belarus	1
Switzerland	11			Israel	3	Comoros	1
Thailand	11			Laos	3	Congo	1
European Union	11			Lesotho	3	North Korea	1
Austria	10			Niger	3	Djibouti	1
Chile	10			Panama	3	El Salvador	1
Egypt	10			Dominican Republic	3	Eritrea	1
Philippines	10			Sri Lanka	3	Gabon	1
Finland	10					Georgia	1
Ghana	10					Guatemala	1
Mexico	10					Guinea-Bissau	1
Nigeria	10					Equatorial Guinea	1
Poland	10					Kiribati	1
Singapore	10					Latvia	1
Sudan	10					Lebanon	1
Zimbabwe	10					Macedonia	1
Costa Rica	9					Malawi	1
						Mozambique	1
						Mongolia	1
						Oman	1
						Papua New Guinea	1
						Pakistan	1
						Central African Republic	1
						Czech Republic	1
						Saint Kitts and Nevis	1
						Sierra Leone	1
						Swaziland	1
						South Sudan	1
						Uzbekistan	1

1 The Codex Committee on Nutrition and Foods for Special Dietary Uses.

a Members attending at least nine sessions.

b Members attending 6–8 sessions.

c Members attending 3–5 sessions.

d Members attending two sessions.

**Figure 1:**
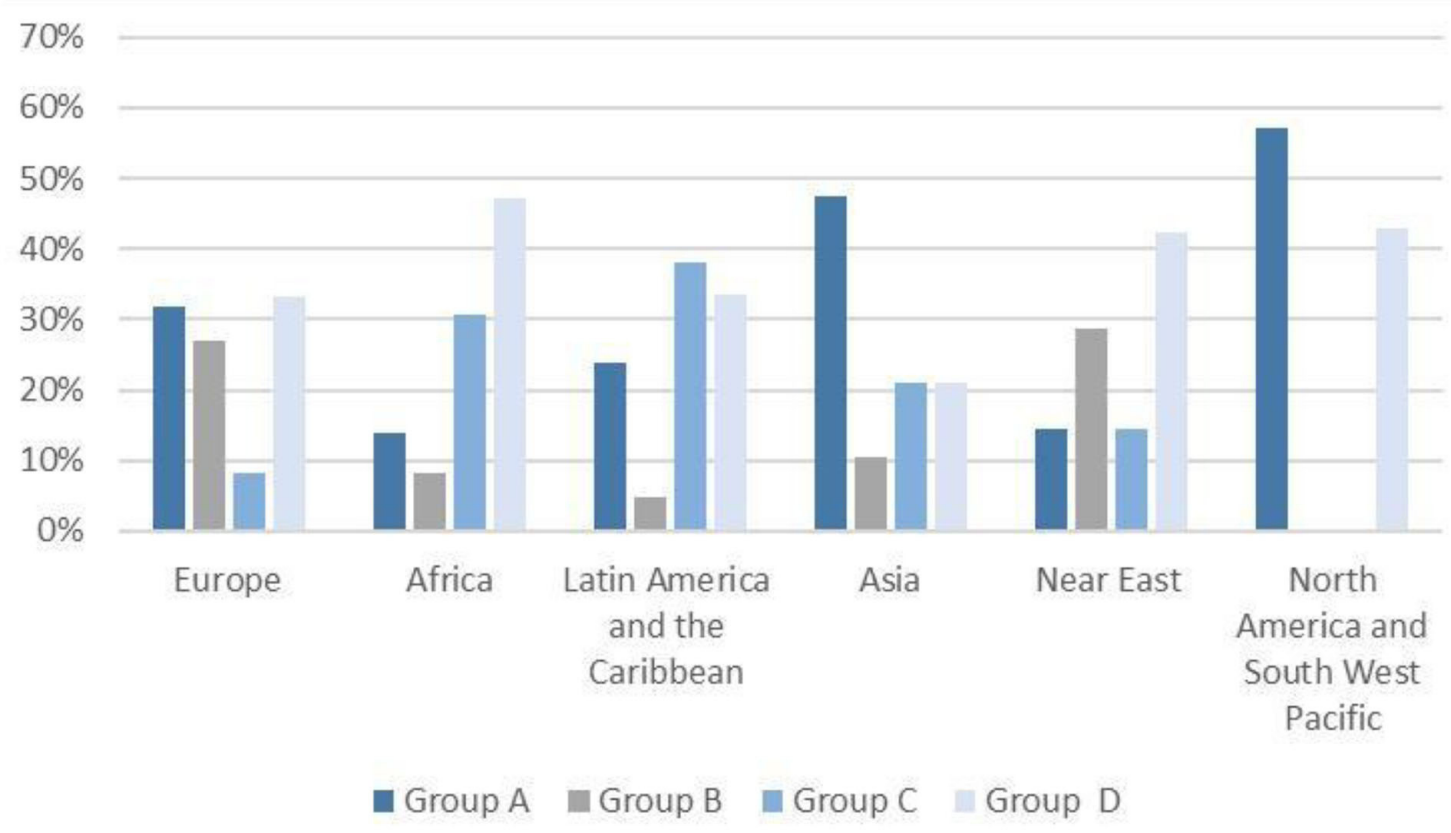
Percentage of participants attending CCNFSDU^a^ sessions by region, 2009–2019. ^a^The Codex Committee on Nutrition and Foods for Special Dietary Uses.

Participation in the review of the Codex was analyzed according to region. The countries in the standard review were divided into regions according to the Codex. Upon observation, it was found that 57.2% of North America and southwest Pacific as well as 47.4% of Asian countries engaged in most parts of the investigated process (Group A). Furthermore, 47.2% of the African countries, 42.4% of the Near Eastern countries, and 33.4% Latin American and Caribbean countries attended < 3 sessions (Group D; Figure 1). On the contrary, 58.7% of European countries participated in at least six of the 11 meetings. Table 4 shows private interest participants percentages (associations, business organizations, and food industry) according to the delegate composition of member countries. Only seven of the 37 Group A members (i.e., South Africa, Belgium, Sweden, Finland, Singapore, Zimbabwe, and the EU) showed a delegation composition with no private sector participation (i.e., private sector accounted for 81% of the delegation members). In the aforementioned group, the French delegation exhibited the highest participation average ratio in the private sector (72.8%), followed by the German (68.2%), the Colombian (63.9%), and the Swiss (59.4%) delegation. Group D did not have any private sector representatives, except for the Congo delegation participating in 2009.

**Table 4:** Private interest participants rate in member countries delegations attending CCNFSDU^a^, 2009–2019.

**Member states**	**Years**											
	**2009**	**2010**	**2011**	**2012**	**2013**	**2014**	**2015**	**2016**	**2017**	**2018**	**2019**	**Average**
	**(%)**	**(%)**	**(%)**	**(%)**	**(%)**	**(%)**	**(%)**	**(%)**	**(%)**	**(%)**	**(%)**	**(%)**
**Group A**												
South Africa	0.0	0.0	0.0	0.0	0.0	0.0	0.0	0.0	0.0	0.0	0.0	0.0
Germany	66.7	60.0	76.9	71.4	78.6	60.0	77.8	66.7	57.1	63.6	71.4	68.2
Australia	0.0	0.0	0.0	50.0	33.3	66.7	66.7	75.0	50.0	50.0	25.0	52.1
Belgium	0.0	0.0	0.0	0.0	0.0	0.0	0.0	0.0	0.0	0.0	0.0	0.0
Brazil	50.0	42.9	60.0	50.0	50.0	50.0	20.0	20.0	16.7	16.7	40.0	37.8
Canada	0.0	0.0	0.0	0.0	0.0	0.0	33.3	40.0	0.0	0.0	0.0	36.7
China	27.3	41.2	47.1	38.9	50.0	45.0	5.9	5.5	13.3	38.0	58.3	33.7
Colombia	50.0	33.3	100	0.0	0.0	100.0	50.0	0.0	50.0	0.0	0.0	63.9
South Korea	0.0	16.7	16.7	20.0	16.7	20.0	0.0	0.0	0.0	0.0	0.0	18.0
United States of America	33.3	36.4	30.8	30.8	33.3	28.6	33.3	23.1	23.5	29.4	26.7	29.9
France	75.0	80.0	75.0	80.0	66.7	50.0	80.0	75.0	75.0	71.4	0.0	72.8
Netherlands	50.0	50.0	50.0	50.0	50.0	0.0	0.0	0.0	0.0	0.0	0.0	50.0
India	33.3	25.0	0.0	0.0	0.0	0.0	0.0	0.0	0.0	0.0	0.0	29.2
Indonesia	0.0	0.0	0.0	14.3	33.3	4.9	57.1	60.0	75.0	42.9	50.0	42.2
Italy	25.0	0.0	0.0	0.0	0.0	0.0	0.0	0.0	0.0	0.0	0.0	25.0
Japan	33.3	16.7	14.3	16.7	16.7	16.7	14.3	0.0	16.7	0.0	0.0	18.2
Kenya	0.0	0.0	0.0	0.0	0.0	50.0	50.0	50.0	33.3	16.7	33.3	38.9
Malaysia	0.0	0.0	50.0	0.0	0.0	72.7	25.0	50.0	25.0	25.0	42.9	41.5
Norway	33.3	33.3	33.3	33.3	0.0	33.3	33.3	0.0	0.0	0.0	0.0	33.3
New Zealand	0.0	0.0	0.0	33.3	50.0	50.0	60.0	60.0	50.0	60.0	50.0	51.7
Sweden	0.0	0.0	0.0	0.0	0.0	0.0	0.0	0.0	0.0	0.0	0.0	0.0
Switzerland	75.0	75.0	66.7	50.0	40.0	50.0	33.3	33.3	80.0	75.0	75.0	59.4
Thailand	40.0	25.0	33.3	33.3	16.7	33.3	50.0	50.0	20.0	50.0	50.0	36.5
European Union	0.0	0.0	0.0	0.0	0.0	0.0	0.0	0.0	0.0	0.0	0.0	0.0
Austria	50.0	0.0	0.0	0.0	0.0	0.0	–	0.0	0.0	0.0	0.0	50.0
Chile	33.3	61.5	0.0	–	0.0	0.0	0.0	50.0	50.0	33.3	0.0	45.6
Egypt	66.7	–	25.0	25.0	0.0	0.0	66.7	60.0	50.0	83.3	75.0	56.5
Philippines	0.0	0.0	0.0	0.0	0.0	–	0.0	50.0	50.0	33.3	33.3	41.7
Finland	0.0	0.0	0.0	–	0.0	0.0	0.0	0.0	0.0	0.0	0.0	0.0
Ghana	0.0	0.0	14.3	0.0	0.0	0.0	0.0	–	0.0	0.0	0.0	14.3
Mexico	66.7	85.7	100.0	33.3	33.3	33.3	40.0	28.6	85.7	57.1	–	56.4
Nigeria	0.0	0.0	20.0	0.0	–	14.3	0.0	0.0	0.0	20.0	0.0	18.1
Poland	0.0	0.0	0.0	0.0	0.0	–	0.0	0.0	33.3	0.0	0.0	33.3
Singapore	0.0	–	0.0	0.0	0.0	0.0	0.0	0.0	0.0	0.0	0.0	0.0
Sudan	0.0	–	0.0	0.0	0.0	0.0	0.0	0.0	0.0	100.0	0.0	100.0
Zimbabwe	–	0.0	0.0	0.0	0.0	0.0	0.0	0.0	0.0	0.0	0.0	0.0
Costa Rica	0.0	50.0	0.0	–	0.0	–	0.0	0.0	0.0	0.0	0.0	50.0
**Group B**												
Saudi Arabia	0.0	–	–	0.0	0.0	0.0	0.0	–	0.0	0.0	0.0	0.0
Spain	0.0	0.0	0.0	0.0	0.0	0.0	0.0	–	–	–	0.0	0.0
Hungary	0.0	–	0.0	0.0	0.0	–	0.0	0.0	0.0	–	0.0	0.0
Ireland	–	–	–	0.0	0.0	0.0	0.0	0.0	0.0	0.0	0.0	0.0
Russia	–	–	–	80.0	66.7	80	83.3	80.0	50.0	33.3	60.0	66.7
Turkey	0.0	–	33.3	0.0	–	0.0	0.0	50.0	0.0	–	25.0	36.1
Vietnam	–	–	–	25.0	0.0	14.3	0.0	33.3	77.7	75.0	35.0	43.4
Algeria	–	0.0	–	0.0	–	0.0	0.0	0.0	0.0	–	0.0	0.0
Denmark	–	0	–	20.0	–	–	33.3	50.0	33.3	50.0	50.0	39.4
Estonia	0.0	–	–	0.0	0.0	–	–	0.0	0.0	0.0	0.0	0.0
Iran	0.0	–	–	0.0	0.0	0.0	0.0	–	–	0.0	0.0	0.0
Lithuania	0.0	–	0.0	0.0	0.0	–	–	–	0.0	0.0	0.0	0.0
Morocco	–	–	0.0	–	–	33.3	0.0	40.0	42.9	40.0	14.3	34.1
Senegal	–	–	–	–	0.0	0.0	0.0	0.0	0.0	0.0	0.0	0.0
Togo	–	–	0.0	0.0	0.0	0.0	0.0	0.0	0.0	–	–	0.0
Argentina	0.0	33.3	–	–	0.0	–	–	–	0.0	25.0	50.0	36.1
Slovakia	–	–	–	–	–	0.0	0.0	0.0	0.0	0.0	0.0	0.0
Kuwait	0.0	–	–	–	0.0	–	0.0	–	0.0	0.0	0.0	0.0
Nepal	–	–	0.0	–	–	–	0.0	0.0	0.0	0.0	0.0	0.0
United Kingdom	0.0	–	0.0	–	–	–	–	0.0	0.0	0.0	0.0	0.0
**Group C**												
Bangladesh	0.0	–	–	–	–	0.0	66.7	–	–	0.0	0.0	66.7
Cameroon	–	–	0.0	0.0	0.0	–	–	–	–	0.0	0.0	0.0
Cambodia	0.0	–	–	–	–	–	0.0	–	0.0	0.0	0.0	0.0
Ivory Coast	0.0	–	–	0.0	0.0	–	–	–	0.0	33.3	–	33.3
Cuba	–	–	–	0.0	–	–	0.0	–	0.0	0.0	0.0	0.0
Ecuador	–	–	–	–	–	–	0.0	100.0	0.0	0.0	0.0	100.0
Mali	–	–	0.0	–	–	–	0.0	–	0.0	0.0	0.0	0.0
Paraguay	0.0	–	–	–	–	–	0.0	0.0	–	0.0	0.0	0.0
Peru	–	0.0	–	–	–	–	0.0	0.0	–	0.0	33.3	33.3
Uganda	–	–	–	–	0.0	–	0.0	0.0	50.0	50.0	–	50.0
Burkina Faso	0.0	–	–	–	–	–	–	–	0.0	0.0	0.0	0.0
Qatar	–	–	0.0	0.0	0.0	–	–	–	0.0	–	–	0.0
Tanzania	–	–	–	–	0.0	0.0	–	–	–	0.0	0.0	0.0
Uruguay	–	0.0	–	–	–	–	0.0	0.0	–	0.0	–	0.0
Benin	0.0	–	0.0	0.0	–	–	–	–	–	–	–	0.0
Bolivia	0.0	0.0	0.0	–	–	–	–	–	–	–	–	0.0
Botswana	–	–	0.0	0.0	–	–	–	–	–	–	0.0	0.0
Croatia	0.0	–	–	–	–	–	–	–	–	0.0	0.0	0.0
Ethiopia	0.0	0.0	0.0	–	–	–	–	–	–	–	–	0.0
Greece	0.0	–	–	–	–	–	–	–	0.0	0.0	–	0.0
Iraq	0.0	–	0.0	–	0.0	–	–	–	–	–	–	0.0
Israel	0.0	–	0.0	–	0.0	–	–	–	–	–	–	0.0
Laos	–	–	–	–	–	33.3	–	–	–	0.0	0.0	33.3
Lesotho	–	–	0.0	0.0	–	–	–	0.0	–	–	–	0.0
Niger	0.0	–	–	–	–	–	–	–	–	0.0	0.0	0.0
Panama	–	0.0	–	–	–	–	–	0.0	–	–	0.0	0.0
Dominican Republic	–	0.0	0.0	–	0.0	–	–	–	–	–	–	0.0
Sri Lanka	–	–	50.0	–	–	–	–	–	0.0	0.0	–	50.0
**Group D**												
Angola	–	–	–	–	–	0.0	–	–	0.0	–	–	0.0
Antigua and Barbuda	–	0.0	–	0.0	–	–	–	–	–	–	–	0.0
Bulgaria	–	0.0	–	–	–	–	–	–	0.0	–	–	0.0
Kazakhstan	–	–	–	–	–	–	–	–	–	0.0	0.0	0.0
Gambia	–	–	0.0	–	–	–	–	–	–	–	0.0	0.0
Yemen	–	–	–	–	0.0	0.0	–	–	–	–	–	0.0
Jamaica	–	0.0	–	–	–	–	–	–	–	0.0	–	0.0
Jordan	–	–	0.0	–	–	–	–	–	–	–	0.0	0.0
Libya	0.0	–	–	0.0	–	–	–	–	–	–	–	0.0
Luxembourg	–	–	–	–	–	0.0	0.0	–	–	–	–	0.0
Mauritania	–	–	0.0	–	0.0	–	–	–	–	–	–	0.0
Moldavia	–	–	0.0	0.0	–	–	–	–	–	–	–	0.0
Burma	–	–	0.0	0.0	–	–	–	–	–	–	–	0.0
Nicaragua	–	–	0.0	–	0.0	–	–	–	–	–	–	0.0
Rwanda	–	–	0.0	–	–	0.0	–	–	–	–	–	0.0
Samoa	0.0	–	0.0	–	–	–	–	–	–	–	–	0.0
Trinidad and Tobago	–	0.0	–	–	0.0	–	–	–	–	–	–	0.0
Tunisia	–	–	–	0.0	0.0	–	–	–	–	–	–	0.0
Armenia	–	–	–	0.0	–	–	–	–	–	–	–	0.0
Azerbaijan	–	–	–	–	–	–	–	–	–	0.0	–	0.0
Belarus	–	–	–	–	–	–	0.0	–	–	–	–	0.0
Comoros	–	0.0	–	–	–	–	–	–	–	–	–	0.0
Congo	100.0	–	–	–	–	–	–	–	–	–	–	100.0
North Korea	0.0	–	–	–	–	–	–	–	–	–	–	0.0
Djibouti	–	–	–	–	–	–	0.0	–	–	–	–	0.0
El Salvador	–	–	–	–	0.0	–	–	–	–	–	–	0.0
Eritrea	0.0	–	–	–	–	–	–	–	–	–	–	0.0
Gabon	–	–	–	0.0	–	–	–	–	–	–	–	0.0
Georgia	–	–	–	–	–	–	–	–	–	0.0	–	0.0
Guatemala	–	–	–	–	–	–	–	–	–	0.0	–	0.0
Guinea-Bissau	–	–	0.0	–	–	–	–	–	–	–	–	0.0
Equatorial Guinea	–	–	–	–	–	–	0.0	–	–	–	–	0.0
Kiribati	0.0	–	–	–	–	–	–	–	–	–	–	0.0
Latvia	–	–	–	–	–	–	0.0	–	–	–	–	0.0
Lebanon	–	–	–	–	–	–	–	–	0.0	–	–	0.0
Macedonia	–	–	–	–	0.0	–	–	–	–	–	–	0.0
Malawi	–	0.0	–	–	–	–	–	–	–	–	–	0.0
Mozambique	0.0	–	–	–	–	–	–	–	–	–	–	0.0
Mongolia	0.0	–	–	–	–	–	–	–	–	–	–	0.0
Oman	–	–	0.0	–	–	–	–	–	–	–	–	0.0
Papua New Guinea	–	–	–	–	–	–	–	–	–	–	–	0.0
Pakistan	–	–	–	0.0	–	–	–	–	–	–	–	0.0
Central African Republic	–	–	–	0.0	–	–	–	–	–	–	–	0.0
Czech Republic	0.0	–	–	–	–	–	–	–	–	–	–	0.0
Saint Kitts and Nevis	–	0.0	–	–	–	–	–	–	–	–	–	0.0
Sierra Leone	0.0	–	–	–	–	–	–	–	–	–	–	0.0
Swaziland	–	–	–	–	0.0	–	–	–	–	–	–	0.0
South Sudan	–	–	–	–	–	–	–	–	–	–	0.0	0.0
Uzbekistan	–	–	–	–	0.0	–	–	–	–	–	–	0.0
**Private interest participants rate**	37.3	44.0	40.3	42.1	43.1	40.9	43.1	42.7	43.6	42.7	41.8	42.0

a The Codex Committee on Nutrition and Foods for Special Dietary Uses.

–, Member state has not attended the meeting.

After observing the total CCNFSDU participants based on the type of interest (including country delegations and observer members), this study found that, in every year, the proportion of public interest institution representatives was greater than the proportion of private interest institution representatives. During the period investigated, the participant average for private interests was 42%, while the average for public interest was 58% (Table 5).

**Table 5:** Total number of participants (including member state delegations and observer delegations) by interest type attending CCNFSDU^1^, 2009–2019.

	**Participants**				
**Years**	**Public interest^a^**		**Private interest^b^**		**Total**
	***n***	**%**	***n***	**%**	
2009	158	63	94	37	252
2010	131	56	103	44	234
2011	160	60	108	40	268
2012	158	58	115	42	273
2013	148	57	112	43	260
2014	176	59	122	41	298
2015	164	57	124	43	288
2016	168	57	125	43	293
2017	177	56	137	44	314
2018	220	57	164	43	384
2019	213	58	153	42	366
Average	170	58	123	42	294

1 The Codex Committee on Nutrition and Foods for Special Dietary Uses.

a Government representatives, universities, scientific organizations, defense of rights organizations, humanitarian organizations, intergovernmental organizations, and consumer organizations.

b Business associations and food industry representatives.

**Figure 2:**
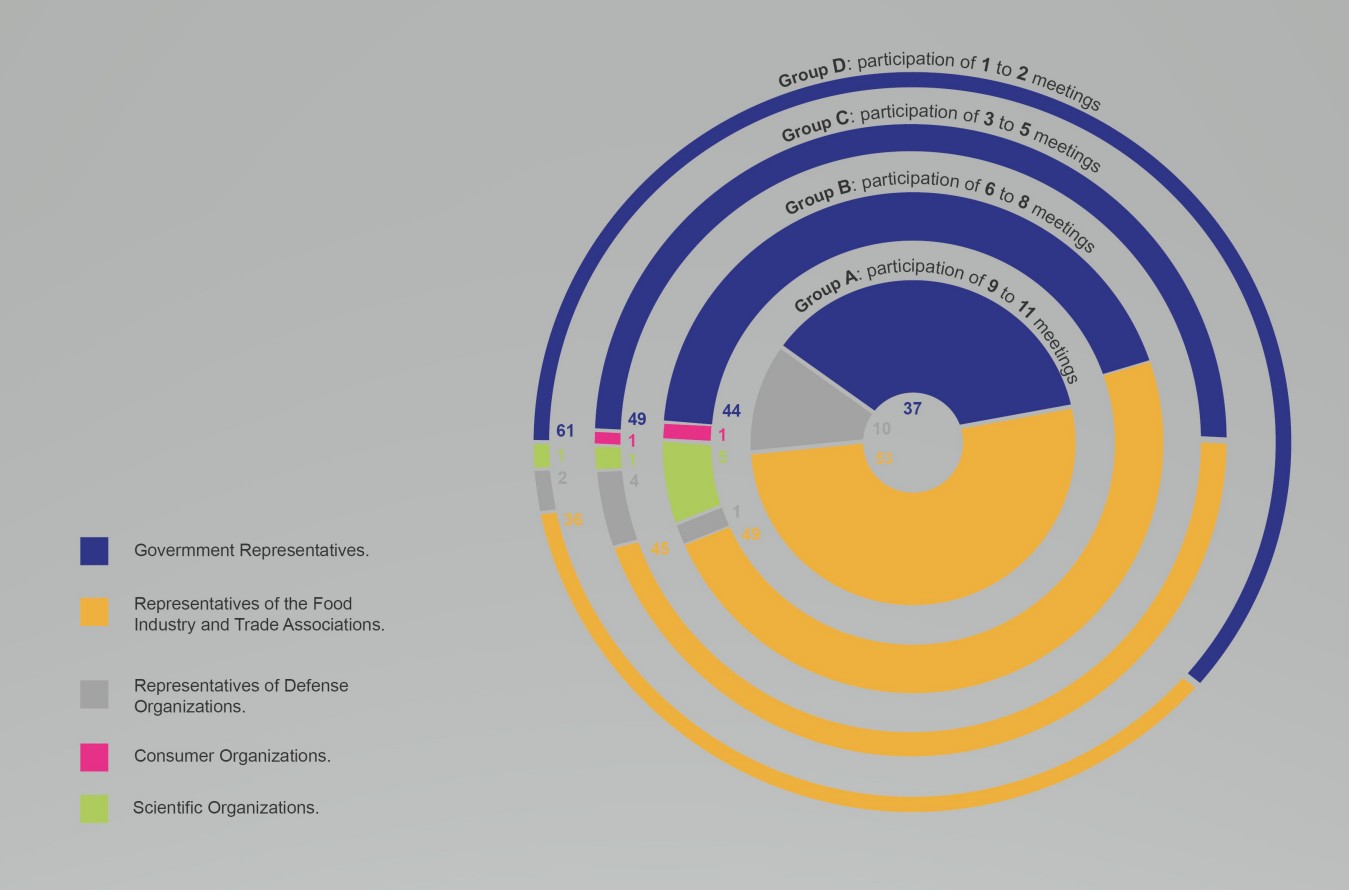
The relative participation of actors according to interest group, attending CCNFSDU^a^, 2009–2019. ^a^The Codex Committee on Nutrition and Foods for Special Dietary Uses.

Figure 2 presents relative participation according to the interest group type and the meeting attendance categories. A lower attendance of the member country in meetings was directly correlated with lower relative participation of representatives of private interests and greater relative participation of government representatives. However, this results in increased relative participation of government representatives. More than half of the participants in the member state delegations in Group A represented business associations and the food industry and 37% were government representatives, while in Group D these proportions were 36 and 61%, respectively.

### The various participations of the baby food industry

**Figure 3:**
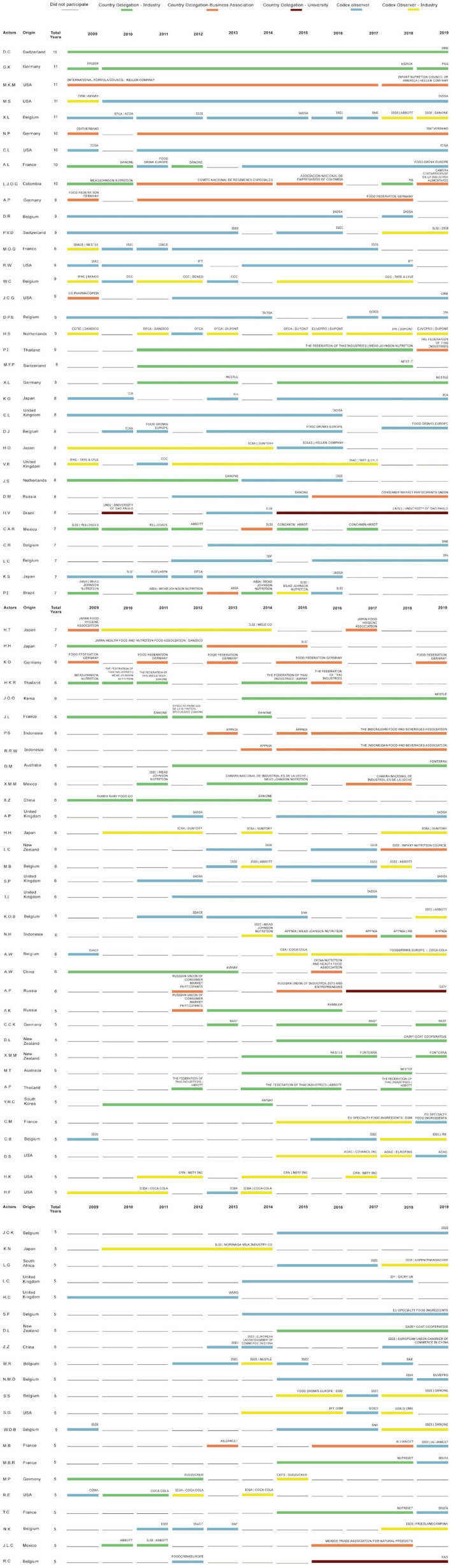
Private interest participants and participating institutions according to the origin and total years attending CCNFSDU^a^, 2009–2019. ^a^Codex Committee on Nutrition and Foods for Special Dietary Uses.

Figure 3 shows the private interest participants and the respective annual representative institutions for Groups A, B, and C. In terms of delegation countries, a trend has been observed of representatives remaining within the same industry or business association, in contrast to representatives of observer organizations, which, in several cases, switch organizations and industries. These participants often change sides, and they sometimes serve as participants for country delegations in the food industry or as an observer organization. In this figure, we can see that three out of the five private interest actors who attended the entire review process represented supply manufacturing industries, such as vitamins, artificial flavors, and nutraceuticals for food industry formulations, as well as analytical and instrumental tests for food safety analysis, such as DSM and Merck.

During the investigated period, the participation of 67 observer organizations was analyzed. Based on the analysis of these organizations, 59.7% (*n* = 40) were business associations advocating food industry interests, 19.4% (*n* = 13) were scientific organizations, 6.0% (*n* = 4) were human rights-based organizations (human and breastfeeding), 4.5% (*n* = 4) were consumer organizations, 7.5% (*n* = 5) were humanitarian organizations, and 3.0% (*n* = 2) were intergovernmental organizations (Table 6).

**Table 6:** Observer organizations attending CCNFSDU^a^ 2009–2019.

**Observer organizations (*n* = 67)**	***N*# Meetings**
**Business associations (*****n*** **=** **40; 59.7% of observer organizations)**	
Council for Responsible Nutrition—CRN	11
International Alliance of Dietary/Food Supplement Associations—IADSA	11
International Council of Beverages Associations—ICBA	11
International Dairy Federation—IDF	11
International Life Sciences Institute—ILSI	11
International Special Dietary Foods Industries—ISDI	11
International Chewing Gum Association—ICGA	10
International Council of Grocery Manufacturers Associations—ICGMA	10
European Dietetic Food Industry Association—IDACE^b^ (currently Specialized Nutrition Europe—SNE)	10
Calorie Control Council—CCC	9
European Food and Drink Industry—Food Drink Europe	9
Institute of Food Technologists—IFT	9
Federation of European Specialty Food Ingredients Industries—EU Specialty Food Ingredients	8
International Council on Amino Acid Science—ICAAS	8
European Association of Sugar Manufacturers—CEFS	7
International Co-operative Alliance—ICA	7
International Food Additives Council—IFAC	7
Association Européenne pour le Droit de L'alimentation/European Food Law Association—EFLA_AEDA	6
International Fruit and Vegetable Juice Association—IFU	6
European Vegetable Protein Association—EUVEPRO	5
International Probiotics Association—IPA	5
Global Organization for EPA and DHA omega-3—GOED	4
Organization des Fabricants de produits Cellulosiques Alimentaires—OFCA	4
Association of the European Self-Medication Industry—AESGP	3
Association for International Promotion of Gums—AIPG	3
International Federation of Margarine Associations—IFMA (currently IMACE)	3
European Chemical Industry Council—CEFIC	2
Confederation of the Food and Drink Industries of the EU—CIAA	2
European Federation of Associations of Health Product Manufacturers—EHPM	2
Food Industry Asia—FIA	2
Association of Yogurts & Live Fermented Milks—YLFA	2
Association des Amidonniers et Féculiers—AAF	1
Association of Manufacturers and Formulators of Enzyme Products—AMFEP	1
European Committee for Umami—ECU	1
European Food and Feed Cultures Association—EFFCA	1
European Association of Polyol Producers—EPA	1
European Salt Producers' Association—EUSALT	1
International Glutamate Technical Committee—IGTC	1
International Wheat Gluten Association—IWGA	1
International Ready-to-Use Foods Association^c^ (NUTRISET)—IRUFA	1
**Scientific associations^d^** **(*****n*** **=** **13; 19.4% of participating organizations)**
European Society for Pediatric Gastroenterology Hepatology and Nutrition—ESPGHAN	7
International Food Policy Research Institute—IFPRI	7
Association of Official Analytical Collaboration International—AOAC	5
Early Nutrition Academy—ENA	5
World Sugar Research Organization—WSRO	5
International Association for the Development of Natural Gums—AIDGUM	4
American Oil Chemists' Society—AOCS	3
American Society for Nutrition—ASN	2
United States Pharmacopeial Convention—USP	2
World Public Health Nutrition Association—WPHNA	2
International Association for Cereal Science and Technology—IACST	1
International Organization for Standardization—ISO	1
World Obesity Federation—WOF	1
**Defense of rights associations (*****n*****=** **4; 6.0% of participating organizations)**
International Baby Food Action Network—IBFAN	11
International Lactation Consultant Association—ILCA	10
European Network of Childbirth Associations—ENCA	8
Association of European Coeliac Societies—AOECS	7
**Humanitarian associations^d^** **(*****n*** **=** **5; 7.5% of participating organizations)**
Helen Keller International—HKI	7
United Nations Children's Fund—UNICEF	6
Global Alliance for Improved Nutrition—GAIN	4
Médecins Sans Frontières International—MSF	4
Action Contre la Faim—ACF	1
**Consumer associations (*****n*** **=** **3; 4.5% of participating organizations)**
National Health Federation—NHF	11
International Association of Consumer Food Organizations—IACFO	10
Consumers International—CI	1
**Intergovernmental associations (*****n*** **=** **2; 3.0% of participating organizations)**
Inter-American Institute for Cooperation on Agriculture—IICA	5
União Africana—UA	5

a The Codex Committee on Nutrition and Foods for Special Dietary Uses.

b IDACE had its name replaced with SNE in October 1, 2013.

c ONG IRUFA is registered in the Codex Website as a NGO, but presented data are from Nutriset, responsible for manufacturing Ready-to-Use Therapeutic Food (RUTF) Plumpy'Nut^®^.

d It was not possible to discern the source of funding for some of the scientific and humanitarian organizations that participated in the process. Some of them may have been financed by industries and actually defend their interests.

**Figure 4:**
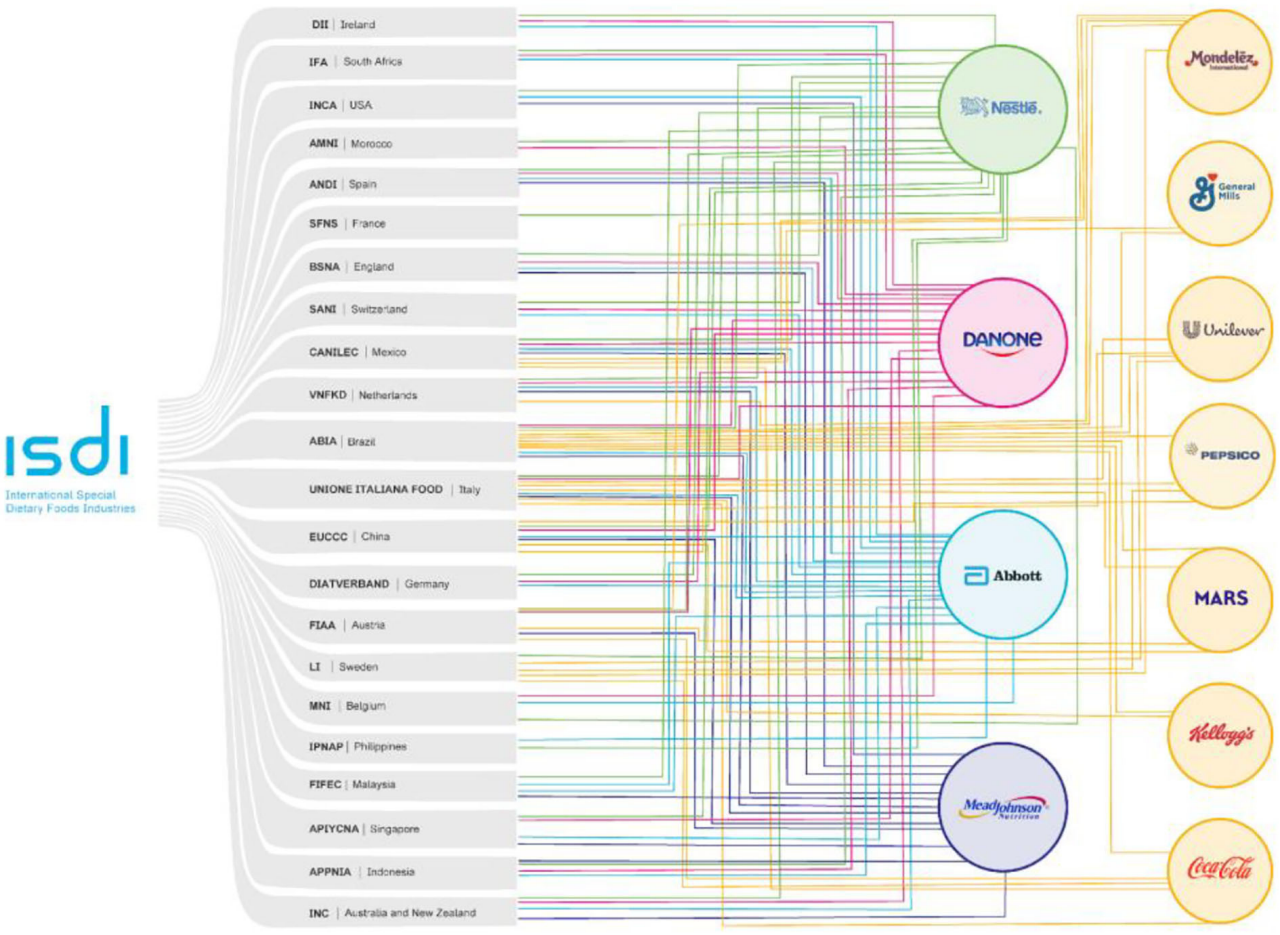
Commercial associations members of International Special Dietary Foods Industries and links with Big Formula.

Considering that the ISDI was the only observer organization that spoke for the interests of the baby food industry during the process of revising the Standard, it was considered important to identify the members of this organization. The study found that Big Formula was an active partner of the ISDI member associations. Nestlé was identified as a member of all ISDI 22 associations, followed by Abbott, with 19 associations, Danone with 17 associations, and MeadJohnson with 13 associations (Figure 4). These results corroborate the literature (13), which indicates that these corporations are structured in a global influence network to protect their interests and promote regulatory environments that benefit their product expansion.

## Discussion

This study identified that 134 of the 189 Codex Alimentarius Commission members (71%) attended the CCNFSDU sessions during the study period (2009–2019). Thus, this indicates different levels of attendance in the countries during the reference period. Smythe (5) underscored that national participation has been relatively unbalanced among the Codex committees despite the recent increase in adhesion among the countries. Furthermore, this unbalance remains despite the WTO's encouragement to its members to standardize their regulations according to the Codex rules and guidelines. Previously, low-income countries experienced challenges attending sessions. Thus, the Codex established a trust fund in 2003 to increase participation in 2004, which financed ~90 representatives from these countries (5). Despite efforts to enhance support, it was found that the participation among middle- and high-income countries was higher. In this sense, understanding the low adherence of these countries to the CCNFSDU is essential to verify whether the Codex norms and guidelines are reflected or not in public policies on infant feeding at the local level.

Industry participation in national delegations was another highlight of the present study which is seemingly common practice in the working processes associated with the Codex. Furthermore, industry participation can serve as a means through which the industry seeks to influence the internal decisions of the delegations. According to Thow et al. (10), this influence results from local lobbyists impacting committees, decision-making authorities, as well as national and global forums (10). However, the inclusion of industry representatives in national delegations led to a further imbalance favoring the industry because observer organizations linked to industry interests are formed exclusively by industry members.

Several authors (5, 7–10) have also registered private interest participants which have compounded member country delegations. Furthermore, non-state actors from corporations and NGOs have played a significant role in the establishment of rules and guidelines through the direct involvement of the Codex Commission and their Committees as well as enhancing national efforts to influence the trading positions of state actors (28). Lee (6) observed an increase in industry participation between 1989 and 1991 on committees dealing with controversial issues, such as the Codex Committee on Food Additives (CCFA), handling additives, and CCNFSDU, handling special foods. The composition of industry actors attending the committee meetings that occurred between 1989 and 1991 was 41 and 47%, respectively (6). These findings are relatively similar to the attendance rates presented in this study (42%). However, the relative participation of the defense of rights, consumers, and scientific organizations was considered insignificant in this study. This participation may have been even smaller, as it was not possible to discern the source of funding for some of the scientific and humanitarian organizations that participated in the process. Some of them may have been financed by industries and actually defend their interests. Previous research (29, 30) has underscored that business groups are repeatedly overrepresented in decision-making politics. In contrast, public interest groups face difficulties in organizing themselves and enabling their participation and thus are underrepresented.

The imbalance observed in this study is consistent with previous literature in terms of the representation of several interest groups. This has resulted in the criticism of the Codex, considering that, in the private sector, this participation can influence food standards to benefit commercial interests (6, 7, 9) instead of public health interests. Throughout the years, representatives of supply manufacturing industries as well as analytical and instrumental tests for food safety analysis integrated Swiss and German national delegations, potentially enhancing technical support and participating in all internal processes. The International Alliance of Dietary/Food Supplement Associations (IADSA) has also participated in the process, representing the interests of dietary and food supplement industries (e.g., Amway, DSM, Merck, Danisco, and Dupont). However, the participation of micronutrient industries only reinforces the debate on nutritional reductionism, which focuses exclusively on nutrients, disregarding the quality of food and the combination of foods that form a dietary pattern (31).

The investigation of participants representing different sectors found that the same participant from the Russian delegation representing the government from 2017 to 2019 also represented the Russian Union of Industrialists and Entrepreneurs in 2016. This union is a non-governmental organization that promotes Russian business community interests. Furthermore, a participant from the Brazilian delegation represented the University of São Paulo in 2010 and from 2015 to 2019. However, from 2012 to 2014, the participant joined the International Life Sciences Institute (ILSI) observer organization delegation. After analyzing this participant's curriculum, it was found that the participant had been an ILSI member since 2012, occupying the position of the Scientific Coordinator at the supposed ILSI Task Force Supplements and Food fortification.

The ILSI was created in 1978 by a former Coca-Cola chief executive officer to prioritize the work of the organization in the scientific and political context. Evidence suggests that the ILSI influences scientific integrity principles through a set of political practices adopted by industry actors to influence public policies, research, and public health practice (32). Several representatives from the ILSI can be identified in state member delegations, such as the Japanese delegation from 2013 to 2015 and the Mexican delegation in 2009, 2011, and 2013, even though this is an observer organization. The same phenomenon is recognized at the ISDI, with participants in the New Zealand delegation in 2018 and 2019 and in the Mexican delegation in 2011.

Upon analyzing the baby food industries in the national delegation, it was found that these industries actively participated in the follow-up formula review. Representatives from Danone were identified in the French, Dutch, Thai, and Chinese delegations. Furthermore, representatives from Nestlé were identified in the Swiss, German, Kenyan, New Zealand, and Australian delegations. Representatives from MeadJohnson were in the Thai, Brazilian, Mexican, Indonesian, and Colombian delegations, while representatives from Abbott were found in the Mexican and Thai delegations. Corporations from the food and pharmaceutical industries of Europe and the USA dominate infant formula manufacturing and account for the major product brands. Moreover, only five of these corporations controlled 57% of the global market share, namely, Nestlé (Switzerland), Danone (France), Reckitt Benckiser (United Kingdom; in 2017 acquired MeadJohnson Nutrition), Abbott Laboratories (USA), and Royal Friesland Campina (Netherlands) (15).

The result of a higher proportion of participation of business associations representing the interests of the food industries among the observer organizations was also reported by Smythe (5). This author noted that there has been an increase in the number of industry-related observer organizations on the Codex over the years. In 1993, 660 out of the 2,578 participants from various Codex Committees represented the industry. Moreover, 140 and 157 observer organizations linked to the industry attended CAC sessions in 1993 and 2007, respectively. In addition, 70% of observer organizations in 2000 and 2002 represented industry interests (5).

Among the 67 observer organizations that attended the CCNFSDU during the evaluation period, only 11 organizations participated in the review of the follow-up formula standard beyond attendance at meetings. Participation comprised performing written comments in response to requests included on Letter Circular (LC) at every review step. Future publications ought to provide detailed information about the documents and the respective observer organizations submitting these documents.

Lauber et al. (33) found that one of the food industry strategies involved in public consultation regulations and specialized agency documents, such as FAO and WHO, is related to business associations. Furthermore, increasing evidence suggests coordination between associations, even though some industry actors are competitors in their respective markets (33). This strategy was also used in this study. All 11 observer organizations effectively involved in standard review through official statements on each consulting document submitted by the CCNFSDU represent a different sector of the baby food industry. Consequently, seven organizations represented specific industries or supply manufacturing industries for formula preparation [e.g., sugar, cow milk, vitamins, minerals, and docosahexaenoic acid (DHA)], namely, the European Association of Sugar Manufacturers (CEFS), American Oil Chemists' Society (AOCS), Institute of Food Technologists (IFT), European Vegetable Protein Association (EUVEPRO), Global Organization for eicosapentaenoic acid (EPA) and DHA omega-3 (GOED), International Dairy Federation (IDF), and Federation of European Specialty Food Ingredients Industries, which is currently referred to as EU Specialty Food Ingredients. Moreover, two organizations, i.e., the International Council of Beverages Associations (ICBA) and ISDI, represented the main food industries on a global scale, while the ISDI was the only organization representing the baby food industry.

The ISDI is a non-profit business association composed of 22 national and local associations that share common goals. Each national association includes various industries. The ISDI comprises the main expert international association representing the special dietary food sector and performs as an industry platform promoting discussion about regulatory, technical, and scientific questions related to special dietary foods. The mission outlined by the ISDI is to support members by ensuring consistent policies based on science (34). Furthermore, the ISDI highlights its role as the Codex “partner,” working as an “official observer at the Codex Alimentarius Commission,” the joint FAO–WHO food standards-setting body (https://www.isdi.org/about). This structure positions the food industry as a legitimate political actor and partner in infant food promotion (33).

An important aspect to be highlighted is the capillarity of the ISDI and the presence of Big Formula industries in almost all associations. In addition to manufacturing and trading various types of both infant and follow-up formula, the industries have economic and political influence and act as a special interest group with the aim of impacting food and nutrition public policies to their benefit. This finding corresponds to the description of Mariah and Martins (30), which states that corporations' organizational capacity and high resource availability provide them a privileged position in relation to public interest groups advocating for collective rights. This is consistent with the case in this study regarding adequate food and health for infants and young children (30).

The results of this study support Baker's et al. (13) hypothesis that Big Formula operates in a global network of business associations and influences organizations. Particularly, Nestlé is a member of most organizations, followed by Danone, MeadJohnson, Abbott, and Friesland Campina (13). This study found that the ISDI business associations' members are located across six continents and are mostly national associations representing the baby food industry and dairy industry interests. Some of these associations gather information about breastfeeding and supplementary food for the general population and healthcare providers, identifying as technical supporters of local governments. In many cases, they pose as civil society organizations of the public interest despite representing corporate interests (35).

Finally, this study observed that food industry interest associations as well as associations focused on infant food are also ISDI members. These include organizations such as Associação Brasileira da Indústria de Alimentos (ABIA) from Brazil, Unione Italiana Food from Italy, Swedish Food Federation from Sweden, European Union Chamber of Commerce from China (EUCCC), and Food Industry Association of Austria (FIAA) from Austria. The largest transnational manufacturers of ultra-processed foods, such as Coca-Cola, Kelloggs, Mars, Pepsico, Unilever, General Mills, and Mondelez, are members of these associations. Representatives from these transnational companies were also identified in the observed delegations, namely, Coca-Cola in the ICBA and Food Drinks Europe, and Kellogs in the ILSI. Moreover, Coca-Cola was part of the US delegation in 2010 and 2011.

This study was limited due to the lack of interviews conducted with participants during the standard follow-up formula review process. This work is exclusively based on official data. Thus, the evidence examined does not provide an in-depth analysis of power discrepancies between participants representing corporations and participants representing public interests. However, this study clearly suggests a predominance of corporations over public interest actors.

The lack of official data on the conflicts of interest of the participants called for the investigation of the profile of each organization, imposing a challenge due to some actors' lack of transparency. Significant efforts have been made to identify the relationship between these organizations and the baby food industries and to overcome underlying limitations. Nevertheless, this relationship may not have been identified in some cases, which may have led to the underestimation of the participation of industries in the analyzed process. The strengths of the study are the thorough examination of all the materials available on each meeting of the CCNFSDU to regulate standards for infant formulas and the synthesis of the findings through infographics, offering the reader a didactic communication resource.

The findings presented here expand the body of evidence confirming the expressive and disproportionate participation of industries and their representatives in the discussion processes within the scope of the CCNFSDU. Few studies have analyzed the Codex and the public documents of their respective committees. Thus, further studies are required to evaluate the internal processes of the Codex and the possible implications for the current regulations. Furthermore, an analysis of empirical data resulting from this study will provide evidence about corporative political action strategies implemented by infant formula industries and associated supplies in the standard for follow-up formula process that adheres to the CCNFSDU. Notably, prior to attending the Codex sessions, there is a preparation of the country's position, for which the articulation of all the actors in the delegation is necessary. As these local articulations are not transparent, we do not know which public interests are compromised and which private interests are benefited in this process. Thus, further studies are required to investigate these processes.

## Data availability statement

The original contributions presented in the study are included in the article/Supplementary material, further inquiries can be directed to the corresponding author.

## Author contributions

KS participated in the study conception, design, data collection, analysis, interpretation, writing, critical revision, and approval of the final version. IC participated in the conception, design, supervision of data collection, analysis, interpretation, writing, critical revision, and approval of the final version. CC participated in the analysis and interpretation, writing, critical revision, and approval of the final version. KC participated in the conception, design, analysis, interpretation, writing, critical revision, and approval of the final version. All authors contributed to the article and approved the submitted version.

## Conflict of interest

The authors declare that the research was conducted in the absence of any commercial or financial relationships that could be construed as a potential conflict of interest.

## Publisher's note

All claims expressed in this article are solely those of the authors and do not necessarily represent those of their affiliated organizations, or those of the publisher, the editors and the reviewers. Any product that may be evaluated in this article, or claim that may be made by its manufacturer, is not guaranteed or endorsed by the publisher.
