# Supplementary material for: Baby food industry interference with infant feeding international regulation—A case study on the standard for follow-up formula
Source: Front Public Health. 2022 Nov 22;10:984385. doi: 10.3389/fpubh.2022.984385 (PMC9723377; doi:10.3389/fpubh.2022.984385)
Supplement: Supplementary file 1 [file Table_1.DOCX]

Description of analyzed documents, CCNFSDU, 1966-2019. (to be continued)

| Session | Year | Document description |
| --- | --- | --- |
| 1^st^ | 1966 | ALINORM 66/4 (1) |
| 2^nd^ | 1967 | ALINORM 68/4 (26) |
| 3^rd^ | 1968 | ALINORM 69/4 (26) |
| 4^th^ | 1969 | ALINORM 70/4 (26) |
| 5^th^ | 1970 | ALINORM 71/4 (26) |
| 6^th^ | 1971 | ALINORM 72/4 (26) |
| 7^th^ | 1972 | ALINORM 74/4 (26) |
| 8^th^ | 1974 | ALINORM 76/4 (26) |
| 9^th^ | 1975 | ALINORM 76/26ª |
| 10^th^ | 1977 | ALINORM 78/4 (26) |
| 11^st^ | 1978 | ALINORM 79/4 (26) |
| 12^nd^ | 1980 | ALINORM 81/4 (26) |
| 13^rd^ | 1982 | ALINORM 83/4 (26) |
| 14^th^ | 1985 | ALINORM 85/4 (26) |
| 15^th^ | 1987 | ALINORM 87/4 (26) |
| 16^th^ | 1988 | ALINORM 89/4 (26) |
| 17^th^ | 1991 | ALINORM 91/4 (26) |
| 18^th^ | 1992 | ALINORM 93/4 (26) |
| 19^th^ | 1995 | ALINORM 95/4 (26) |
| 20^th^ | 1996 | ALINORM 97/4 (26) |
| 21^st^ | 1998 | ALINORM 99/4 (26) |

Description of analyzed documents, CCNFSDU, 1966-2019. (to be continued).

| Session | Year | Document description |
| --- | --- | --- |
| 22^nd^ | 2000 | ALINORM 01/4 (26) |
| 23^rd^ | 2001 | ALINORM 03/4 (26) |
| 24^th^ | 2002 | ALINORM 03/26ª |
| 25^th^ | 2003 | ALINORM 04/27/26 |
| 26^th^ | 2004 | ALINORM 05/28/26 |
| 27^th^ | 2005 | ALINORM 06/29/26 |
| 28^th^ | 2006 | ALINORM 07/30/26 |
| 29^th^ | 2007 | ALINORM 08/31/26 |
| 30^th^ | 2008 | ALINORM 09/32/26 |
| 31^st^ | 2009 | ALINORM 10/33/26 |
| 32^nd^ | 2010 | REP 11/NFSDU |
| 33^rd^ | 2011 | 1.REP12/NFSDU 2.CX/NFSDU 11/33/10: PROPOSAL TO REVIEW THE CODEX STANDARD FOR FOLLOW-UP FORMULA (CODEX STAN 156-1987) - BACKGROUND PAPER- Prepared by New Zealand |
| 34^th^ | 2012 | 1. REP 13/NFSDU |
| 35^th^ | 2013 | 1.REP 14/NFSDU 2.CX/NFSDU 13/35/3: MATTERS OF INTEREST ARISING FROM FAO AND WHO - Prepared by FAO and WHO 3.CX/NFSDU 13/35/7: REVIEW OF THE CODEX STANDARD FOR FOLLOW-UP FORMULA (CODEX STAN 156-1987) -Report of the Electronic Working Group (EWG) Chaired by New Zealand and co-chaired by France and Indonesia |
| 36^th^ | 2014 | 1. REP15/NFSDU 2.CX/NFSDU 14/36/7: REVIEW OF THE STANDARD FOR FOLLOW-UP FORMULA (CODEX STAN 156-1987)(at Step 4)-(Prepared by an EWG led by New Zealand with the assistance of France and Indonesia |

Description of analyzed documents, CCNFSDU, 1966-2019. (to be continued).

| Session | Year | Document description |
| --- | --- | --- |
| 37^th^ | 2015 | 1. REP16/NFSDU 2.CRD 2: PHYSICAL WORKING GROUP REPORT ON REVIEW OF THE STANDARD FOR FOLLOW-UP FORMULA (CODEX STAN 156-1987) - Prepared by New Zealand and France  3.CRD 5: Comments of Colombia, Ecuador, European Union, India, Kenya, Malaysia, Mexico, Vietnam and IBFAN 4.CRD 11: Comments of ISDI 5. CRD 15: Comments of Thailand, ELC and IFT 6. CX/NFSDU 15/37/5 Add.2: Comments of Egypt, Malaysia and Mali 7.CX/NFSDU 15/37/5-Add.1: Comments of Argentina, Brazil, Canada, Chile, Costa Rica, Ghana, Morocco, New Zealand, Norway, Philippines, United States of America, African Union, ELC, ENSA, EUVEPRO, GOED, IDF, IFT and ISDI 8. CX/NFSDU 15/37/5: REVIEW OF THE STANDARD FOR FOLLOW-UP FORMULA (CODEX STAN 156-1987) Prepared by ^th^e Electronic Working Group led by New Zealand with the assistance of France and Indonesia-(At Step 3) |
| 38^th^ | 2016 | 1. REP17/NFSDU 2. CRD 2: REVIEW OF THE STANDARD FOR FOLLOW-UP FORMULA (CODEX STAN 156-1987)-Physical Working Group Report Prepared by the New Zealand and France  3.CRD 3:REVIEW OF THE STANDARD FOR FOLLOW-UP FORMULA -Comments of India, Indonesia, Kenya, Nigeria, African Union 4. CX/NFSDU 16/38/6: REVIEW OF THE STANDARD FOR FOLLOW-UP FORMULA (CODEX STAN 156-1987)-Prepared by the Electronic Working Group led by New Zealand with the assistance of France and Indonesia (At Step 3) 5. CX/NFSDU 16/38/6-Add.1: REVIEW OF THE STANDARD FOR FOLLOW-UP FORMULA (CODEX STAN 156-1987) -Comments of Argentina, Brazil, Canada, Colombia, Costa Rica, Cuba, Nepal, New Zealand, Norway, Philippines, United States of America, AOCS, CEFS, ELC, ENSA, ENSA/EUVEPRO, HKI, IBFAN, IDF and ISDI  6. CX/NFSDU 16/38/6-Add.2: REVIEW OF THE STANDARD FOR FOLLOW-UP FORMULA (CODEX STAN 156-1987)- Comments of Ecuador, the European Union, Malaysia, Thailand, Vietnam and IFT |

Description of analyzed documents, CCNFSDU, 1966-2019. (to be continued).

| Session | Year | Document description |
| --- | --- | --- |
| 39^th^ | 2017 | 1.REP18/NFSDU 2.NFSDU/39 CRD/07: REVIEW OF THE STANDARD FOR FOLLOW-UP FORMULA (CODEX STAN 156-1987)- Comments of Costa Rica, El Salvador, European Union, Kyrgyzstan, Morocco, Nigeria, Sierra Leone, Sri Lanka, African Union and EU Specialty Food Ingredients 3. NFSDU/39 CRD/17: Comments of Indonesia 4. NFSDU/39 CRD/24: Comments of European Vegetable Protein Association (EUVEPRO) - Discussion Paper by Canada and the United States of America for a JEMNU REQUEST FOR THE ESTABLISHMENT OF NITROGEN TO PROTEIN CONVERSION FACTORS FOR SOY AND MILK PROTEINS  5.NFSDU/39 CRD/25: Comments of Mali 6. NFSDU/39 CRD/26: Comments of Mexico 7.NFSDU/39 CRD/19: Comments of Republic of Korea 8. CX/NFSDU 17/39/4 Rev.1: REVIEW OF THE STANDARD FOR FOLLOW-UP FORMULA (CODEX STAN 156-1987)(Prepared by the electronic working group led by New Zealand, France and Indonesia) 9. CX/NFSDU 17/39/4-Add.1: Review of the Standard for Follow-Up-Formula (CODEX STAN 156-1987)- Comments at Step 3 (Replies to CL 2017/75-NFSDU)-Comments of Argentina, Brazil, Colombia, Ecuador, India, Japan, Nepal, New Zealand, Russia, Senegal, Switzerland, United States of America, EU Speciality Food Ingredients, Global Organization for EPA and DHA Omega-3s (GOED), Helen Keller International (HKI), International Association of Consumer Food Organizations (IACFO), International Baby Food Action Network (IBFAN), International Dairy Federation (IDF), Institute of Food Technologists (IFT), International Special Dietary Foods Industries (ISDI) and UNICEF 10.CX/NFSDU 17/39/4-Add.2: REVIEW OF THE STANDARD FOR FOLLOW-UP-FORMULA (CODEX STAN 156-1987)-Comments of Australia, Canada, Kenya, Malaysia, Norway, Philippines, Tanzania, Thailand, European Food Law Association (EFLA), International Council of Grocery Manufacturer Associations (ICGMA), International Special Dietary Foods Industries (ISDI) |

Description of analyzed documents, CCNFSDU, 1966-2019. (to be continued).

| Session | Year | Document description |
| --- | --- | --- |
| 40^th^ | 2018 | 1. REP19/NFSDU 2. NFSDU/40 CRD/5: Editorial and technical amendments to the Essential Composition Requirements for Follow-up Formula for older infants and [Name of Product] for young children-(Prepared by the Chair of the EWG of the review of the Standard for Follow-Up Formula) 3.NFSDU/40 CRD 6: DRAFT REVIEW OF ^TH^E STANDARD FOLLOW-UP FORMULA: ESSENTIAL COMPOSITION REQUIREMENTS Comments at Step 6-Comments of ISDI  4. NFSDU/40 CRD 15: REVIEW OF THE STANDARD FOR FOLLOW-UP FORMULA (CXS 156-1987): SCOPE, PRODUCT DEFINITION, LABELLING- Comments of IACFO  5.NFSDU/40 CRD 23: Comments of India 6. NFSDU/40 CRD 24: REVIEW OF THE STANDARD FOR FOLLOW-UP FORMULA (CXS 156-1987): SCOPE, PRODUCT DEFINITION, LABELLING - Comments of EFLA  7. NFSDU/40 CRD 25: Comments of the African Union 8.NFSDU/40 CRD 29:Comments of the Russian Federation 9. NFSDU/40 CRD 31: Comments of Mexico 10.CX/NFSDU 18/40/5:REVIEW OF THE STANDARD FOR FOLLOW-UP FORMULA (CXS 156-1987)-(Prepared by the electronic working group led by New Zealand, France and Indonesia) 11. CX/NFSDU 18/40/5-Add.1: Review of the Standard for Follow-up Formula: scope, product definition and labelling Comments at Step 3 in reply to CL 2018/63-NFSDU-Comments of Argentina, Australia, Brazil, Cambodia, Canada, Colombia, Costa Rica, Côte d’Ivoire, Ecuador, Egypt, European Union, Ghana, India, Indonesia, Iran, Jamaica, Malaysia, Mali, Nepal, New Zealand, Norway, Peru, Philippines, Senegal, Sri Lanka, Switzerland, United States of America, Viet Nam, EU Specialty Food Ingredients, HKI, IBFAN, ISDI and UNICEF 12.CX/NFSDU 18/40/4-Rev: REVIEW OF THE STANDARD FOR FOLLOW-UP FORMULA: ESSENTIAL COMPOSITION REQUIREMENTS Comments at Step 6 (Replies to CL 2018/62/OCS-NFSDU)- Comments of Australia, Brazil, Canada, Colombia, Indonesia, Japan, New Zealand, Norway, Peru, Philippines, Switzerland, United States of America, AOCS, EU Specialty Food Ingredients, EUVEPRO and ISDI 13.CX/NFSDU 18/40/4-Add.1: DRAFT REVIEW OF THE STANDARD FOR FOLLOW-UP FORMULA: ESSENTIAL COMPOSITION REQUIREMENTS Comments at Step 6-Comments of Egypt, the European Union, Singapore, United States of America and Vietnam |

Description of analyzed documents, CCNFSDU, 1966-2019. (to be continued).

| Session | Year | Document description |
| --- | --- | --- |
| 41^st^ | 2019 | 1. REP20/NFSDU 2. NFSDU/41 CRD 21: Comments by Republic of Korea 3.NFSDU/41 CRD 3: REVIEW OF THE STANDARD FOR FOLLOW-UP FORMULA: DRAFT SCOPE, DESCRIPTION AND LABELLING FOR FUF FOR OLDER INFANTS-Prepared by New Zealand-Background Paper on Cross Promotion 4. NFSDU/41 CRD 4: REVIEW OF THE STANDARD FOR FOLLOW-UP FORMULA-Prepared by New Zealand-Relevant concepts and technical guidance in WHO/WHA documents and the labelling provisions in the draft standard for follow-up formula 5. CX/NFSDU 19/41/4: REVIEW OF THE STANDARD FOR FOLLOW-UP FORMULA: DRAFT SCOPE, DESCRIPTION AND LABELLING FOR FOLLOW-UP FORMULA FOR OLDER INFANTS Replies to comments at Step 6 to CL 2019/77-NFSDU-Comments of Argentina, Australia, Brazil, Burkina Faso, Cambodia, Canada, Costa Rica, European Union, Ghana, Indonesia, Iran, Iraq, Kuwait, Mali, New Zealand, Nepal, Norway, Peru, Senegal, Somalia, Sri Lanka, Switzerland, United States of America, Vietnam, HKI, ISDI, UNICEF 6.CX/NFSDU 19/41/4-Add.1:REVIEW OF THE STANDARD FOR FOLLOW-UP FORMULA: DRAFT SCOPE, DESCRIPTION AND LABELLING FOR FOLLOW-UP FORMULA FOR OLDER INFANTS Comments of Ecuador, Indonesia, Kenya, Mali, Senegal, HKI, IBFAN, WPHNA  7.CX/NFSDU 19/41/5:REVIEW OF THE STANDARD FOR FOLLOW-UP FORMULA (CXS 156-1987) PROPOSED DRAFT FOLLOW UP FORMULA FOR OLDER INFANTS AND [PRODUCT] FOR YOUNG CHILDREN-Prepared by the Electronic Working Group led by New Zealand, France and Indonesia  8.NFSDU/41 CRD 35:Comments by India 9.NFSDU/41 CRD 45:Comments by Lao People's Democratic Republic 10.NFSDU/41 CRD 41:REVIEW OF THE STANDARD FOR FOLLOW-UP FORMULA: DRAFT SCOPE, DESCRIPTION AND LABELLING FOR FUF FOR OLDER INFANTS-Comments of Malaysia 11.NFSDU/41 CRD 51:Comments by Morocco 12.NFSDU/41 CRD 42: Comments by Mexico 13.NFSDU/41 CRD 27:Comments by Nepal |

Description of analyzed documents, CCNFSDU, 1966-2019. (Conclusion).

| Session | Year | Document description |
| --- | --- | --- |
| 41^st^ | 2019 | 14.NFSDU/41 CRD 32:Comments by Nigeria 15.NFSDU/41 CRD 09:REVIEW OF THE STANDARD FOR FOLLOW-UP FORMULA: ESSENTIAL COMPOSITION REQUIREMENTS FOR FOLLOW-UP FORMULA FOR OLDER INFANTS AND [PRODUCT] FOR YOUNG CHILDREN-Comments by Indonesia, Kenya, Mali, Senegal, HKI  16.NFSDU/41 CRD 10:REVIEW OF THE STANDARD FOR FOLLOW-UP FORMULA (CXS 156-1987): PROPOSED DRAFT PRODUCT DEFINITION AND LABELLING FOR [PRODUCT] FOR YOUNG CHILDREN-Comments by Indonesia, Mali, Philippines, Senegal, HKI, ISDI, WPHNA 17.NFSDU/41 CRD 47:REVIEW OF THE STANDARD FOR FOLLOW-UP FORMULA: PROPOSED DRAFT FOLLOW-UP FORMULA FOR OLDER INFANTS AND [PRODUCT] FOR YOUNG-Comments by Panama 18.NFSDU/41 CRD 33:Comments by the Dominican Republik 19:NFSDU/41 CRD 36:Comments by the Russian Federation 20.NFSDU/41 CRD 25:Comments by Thailand 21.NFSDU/41 CRD 28:Comments by the European Union 22.NFSDU/41 CRD 48:REVIEW OF THE STANDARD FOR FOLLOW-UP FORMULA: DRAFT SCOPE, DESCRIPTION AND LABELLING FOR FUF FOR OLDER INFANTS-Comments by Uruguay |

Source: The author, 2021.
